# Incidence, angiographic and clinical predictors, and impact of stent thrombosis: a 6-year survey of 6,545 consecutive patients

**DOI:** 10.1007/s12471-019-1253-2

**Published:** 2019-03-20

**Authors:** R. Rozemeijer, C. Wing Wong, G. Leenders, L. Timmers, S. Koudstaal, S. Z. Rittersma, A. Kraaijeveld, M. Bots, P. Doevendans, P. Stella, M. Voskuil

**Affiliations:** 1 Department of Cardiology, University Medical Centre Utrecht, Utrecht, The Netherlands; 2 Department of Epidemiology, Julius Centrum, Utrecht, The Netherlands; 3 Netherlands Heart Institute, Utrecht, The Netherlands

**Keywords:** Coronary stent thrombosis, Dual antiplatelet therapy, Drug-eluting stent

## Abstract

**Objective:**

We sought to determine the incidence, angiographic predictors, and impact of stent thrombosis (ST).

**Background:**

Given the high mortality after ST, this study emphasises the importance of ongoing efforts to identify angiographic predictors of ST.

**Methods:**

All consecutive patients with angiographically confirmed ST between 2010 and 2016 were 1:4 matched for (1) percutaneous coronary intervention (PCI) indication and (2) index date ±6 weeks to randomly selected controls. Index PCI angiograms were reassessed by two independent cardiologists. A multivariable conditional logistic regression model was built to identify independent predictors of ST.

**Results:**

Of 6,545 consecutive patients undergoing PCI, 55 patients [0.84%, 95% confidence interval (CI) 0.63–1.10%] presented with definite ST. Multivariable logistic regression identified dual antiplatelet therapy (DAPT) non-use as the strongest predictor of ST (odds ratio (OR) 10.9, 95% CI 2.47–48.5, *p* < 0.001), followed by: stent underexpansion (OR 5.70, 95% CI 2.39–13.6, *p* < 0.001), lesion complexity B2/C (OR 4.32, 95% CI 1.43–13.1, *p* = 0.010), uncovered edge dissection (OR 4.16, 95% CI 1.47–11.8, *p* = 0.007), diabetes mellitus (OR 3.23, 95% CI 1.25–8.36, *p* = 0.016), and residual coronary artery disease at the stent edge (OR 3.02, 95% CI 1.02–8.92, *p* = 0.045). ST was associated with increased rates of mortality as analysed by Kaplan-Meier estimates (27.3 vs 11.3%, *p*_log-rank_ < 0.001) and adjusted Cox proportional-hazard regression (hazard ratio 2.29, 95% CI 1.03–5.10, *p* = 0.042).

**Conclusions:**

ST remains a serious complication following PCI with a high rate of mortality. DAPT non-use was associated with the highest risk of ST, followed by various angiographic parameters and high lesion complexity.

**Electronic supplementary material:**

The online version of this article (10.1007/s12471-019-1253-2) contains supplementary material, which is available to authorized users.

## What’s new?

- We determined the incidence of definite stent thrombosis in a Dutch cohort of percutaneous coronary intervention patients.
- A thorough re-evaluation of over 300 baseline and post-PCI coronary angiograms was performed by two blinded interventional cardiologists, using standardised definitions.
- Dual antiplatelet therapy was evaluated using prescription data that were linked to pharmacy records.
- Comprehensive methodological (i. e. multiple imputation chained equation) and statistical (i. e. multivariable conditional logistic regression model, and covariate adjusted Cox proportional-hazard regression) techniques were evaluated by experts.
- The long-term impact of stent thrombosis (up to 5 years) on mortality was evaluated.

## Introduction

Despite substantial improvements in percutaneous coronary intervention (PCI) with novel stent platforms and more potent P2Y12 inhibitors, coronary stent thrombosis (ST) remains a harmful complication with high rates of mortality [1]. Previous reports investigating risk factors for ST were hampered by the rarity of this complication [2] and focused on the clinical profile rather than incorporating a thorough re-evaluation of the coronary angiogram. Hence, we performed a comprehensive analysis on the incidence, angiographic predictors, and clinical impact of ST in a real-world population of patients undergoing PCI.

## Methods

### Study design and study population

A nested case-control study was conducted at our tertiary centre. Consecutive patients with angiographically confirmed ST as defined according to Academic Research Consortium criteria [3] between March 2010 and November 2016 were included in the study. We did not incorporate cases of very late ST (i. e. ST beyond 1 year after PCI). Angiographically confirmed acute, subacute, or late ST cases were matched to controls without ST in a 1:4 ratio based on (1) clinical indication and (2) index date ± 6 weeks of the PCI, and were randomly selected from a consecutive patient cohort of 6,545 patients to comprehensively study the incidence, angiographic predictors, and impact of definite ST. We did not use any restrictions in the random selection of matched controls. The study complied with the Declaration of Helsinki, and explicit Ethics Committee approval from our institution was waived given the non-experimental design of the study.

### PCI and pharmacotherapy

PCI was performed according to standard techniques. Patients were treated with aspirin and a P2Y12 inhibitor prior to PCI, and 70–100 IU/kg of heparin during the procedure. Thereafter, aspirin was continued indefinitely, and the P2Y12 inhibitor was continued as defined by national and international guidelines for a period of 1–12 months: 1 month following bare-metal stents (BMS) and 6–12 months following drug-eluting stents (DES). Implantation of BMS and use of GpIIb/IIIa inhibitors were at the operators’ discretion.

### Reassessment of coronary angiography

Coronary angiography reassessment was conducted using Xcelera software (Philips Healthcare, Best, The Netherlands). Baseline and post-PCI angiograms of the index procedures were individually reassessed in random order by two well-experienced interventional cardiologists who were blinded to the objective of the study and to patient outcome. In case of disagreement, a consensus was reached by consulting a third interventional cardiologist. A complete list of the angiographic definitions used in our study is provided in the Supplementary Appendix. Subsequently, the level of inter-observer reliability [4] (i. e. Cohen’s kappa coefficient, κ) was calculated. Cohen’s kappa coefficient ranges from −1.0 to +1.0, and was considered: poor (κ < 0), slight (κ = 0–0.20), fair (κ = 0.21–0.40), moderate (κ = 0.41–0.60), or substantial (κ = 0.61–0.80). Lesion complexity was defined according to the modified American College of Cardiology/American Heart Association criteria [5] as lesion type B2 or C.

### *Data acquisition*

Detailed clinical, procedural, and angiographic data of the index PCI were obtained for both cases and controls. After hospital discharge, the duration and compliance of antiplatelet and antithrombotic agents were verified from prescription data linked to pharmacy records. To study the use and impact of antiplatelet therapy, we compared dual antiplatelet therapy (DAPT) use at the time of index PCI to angiographically confirmed ST or 30 days prior to ST for cases. Then, we compared this to the same time period for the matched controls. Follow-up data were collected during hospital admissions, routine visits to the outpatient clinic, by a medical questionnaire, and by telephone assessment at 12 months after PCI. Information regarding the patients’ vital status was obtained by the Dutch National Civil Registration.

### Statistical analysis

Continuous variables are reported as means ± standard deviations (SD), and categorical variables as numbers (*n*) and percentages (%) compared using chi-square or Fisher exact test, Student’s *t*-test or Mann-Whitney U test, as appropriate. The cumulative incidence of angiographically confirmed ST with a 95% confidence interval (CI) was calculated using the Wilson method as appropriate for events with low incidence rates. After visually checking the randomness of missing data, multiple imputation chained equation was performed using 5 datasets with 10 iterations [6]. A multivariable conditional logistic regression model was built to identify independent predictors of ST with odd ratios (OR) with 95% CI as a summary statistic. Kaplan-Meier estimates for the risk of death were analysed using a log-rank test, and reported according to good clinical practice statement [7]. An adjusted Cox proportional-hazard regression for the risk of death was performed with adjustment for: age, sex, body-mass index, hypertension, dyslipidaemia, diabetes mellitus, smoking status, left ventricular ejection fraction, estimated glomerular filtration rate, prior acute coronary syndrome, stroke, peripheral artery disease, malignancy, and three-vessel disease. All statistical analyses were performed using SPSS version 25 (SPSS Inc., Chicago, IL, USA), and figures were generated using GraphPad Prism software version 7 (GraphPad, Inc., San Diego, CA, USA).

## Results

**Fig. 1:**
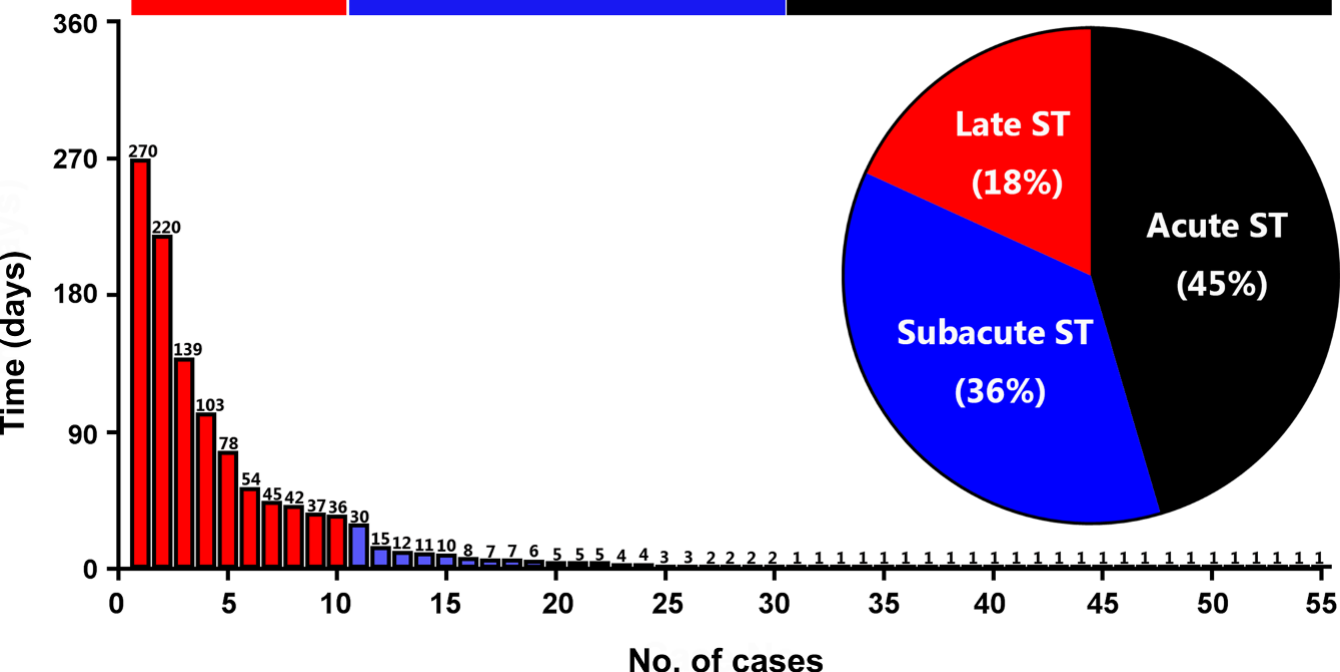
Incidence and timing of angiographically confirmed stent thrombosis (*ST*)

**Fig. 2:**
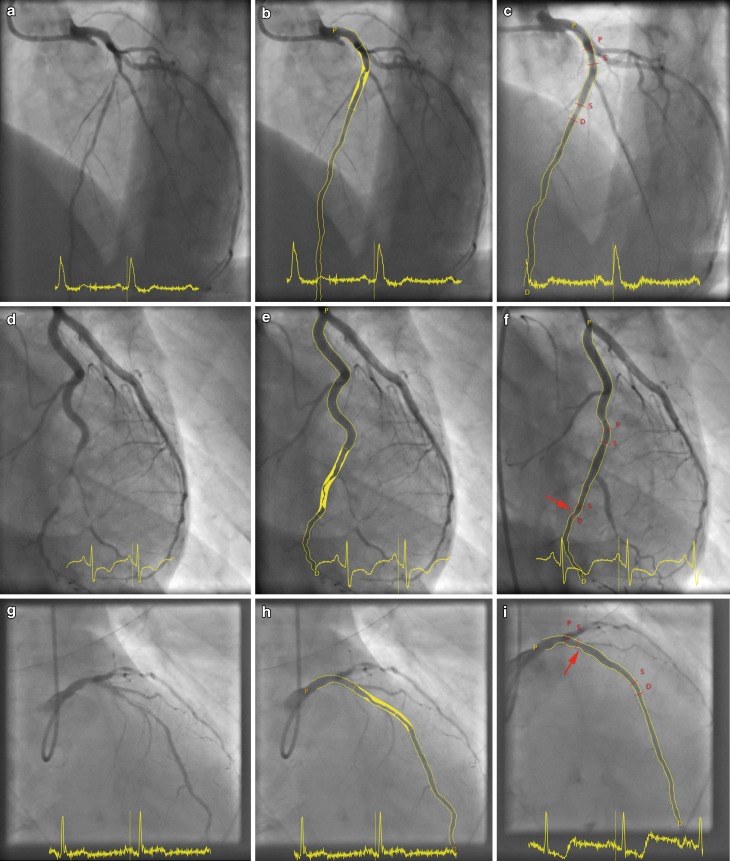
**a**–**i** Coronary angiograms of the target lesion before treatment, during quantitative coronary angiography, and after stent implantation. **a** Bifurcation lesion in the left anterior descending artery, **b** quantified as 21 mm with 70% stenosis, and **c** after implantation of a drug-eluting stent (*DES*). **d** Complex tortuous lesion in the circumflex branch of the left coronary artery, **e** quantified as 24 mm and 90% stenosis; **f** treatment with DES implantation was complicated by an uncovered stent edge dissection on the distal stent edge (*arrow*) that may have induced stent thrombosis (ST). **g** Heavily calcified lesion in the left anterior descending artery, **h** quantified as 29 mm with 70% stenosis, and **i** treated with a DES that appeared to be underexpanded (*arrow*), despite aggressive post-dilatation of up to 28 atm with a non-compliant balloon, which may have triggered ST. *D* Distal segment <5 mm from the stent edge, *P* proximal segment <5 mm from the stent edge, *S* stent edge

From March 2010 to November 2016, a total of 6,545 consecutive patients underwent PCI in our tertiary centre with 10,440 stent implantations. In this period angiographically confirmed ST occurred in 55 patients with a cumulative incidence of 0.84% (95% CI 0.63–1.10%), and decreased over time from 0.89% (95% CI 0.64–1.24%) in 2010–2013 to 0.76% (95% CI 0.50–1.18%) in 2013–2016. Regarding implanted stent type, ST was related to BMS in 26 of the 2,204 patients (1.19%) as compared to 29 of the 4,341 patients (0.67%) that received DES (*p* = 0.032). The timing of ST was acute in 25 patients (46%), subacute in 20 patients (36%), and late in 10 patients (18%) (Fig. 1). Detailed information on baseline characteristics is shown in Tab. 1. Patients who suffered ST had insulin-dependent diabetes more often, had a history of PCI or acute coronary syndrome more often, and significantly more severe left ventricular dysfunction. Procedural characteristics are shown in Tab. 2. We found significantly more cardiovascular risk factors, and complex lesion characteristics (Fig. 2) in patients with ST.

**Table 1:** Baseline demographics and clinical indication for percutaneous coronary intervention in patients with angiographically confirmed stent thrombosis and matched controls

	Angiographically confirmed ST (*n* = 55)	Matched controls (*n* = 220)	*p*-value
*Baseline demographics*			
Age, years	64.9 ± 11.5	65.6 ± 14.7	0.68
Male sex	43 (78.2)	167 (75.9)	0.44
BMI, kg/m^2^	27.3 ± 4.4	26.7 ± 4.5	0.47
Hypertension	29 (52.7)	111 (51.8)	0.86
Dyslipidaemia	27 (49.1)	91 (41.3)	0.31
Diabetes mellitus	16 (29.1)	40 (18.2)	0.072
IDDM	12 (21.8)	19 (8.6)	0.005
Estimated GFR, <60 ml/min/1.73m^2^	10 (18.2)	44 (20.0)	0.39
Current smoker	23 (41.8)	77 (35.0)	0.36
Positive family history	24 (43.6)	77 (35.2)	0.24
Prior PCI	29 (52.7)	24 (43.6)	<0.001
Prior ACS	23 (41.8)	45 (20.5)	<0.001
Prior stroke	3 (5.5)	10 (4.5)	0.77
Prior PAD	6 (10.9)	21 (9.5)	0.76
Prior malignancy	9 (4.1)	2 (3.6)	0.87
LVEF <35%	9 (10.9)	9 (4.1)	0.046
Three-vessel disease	12 (21.8)	26 (11.8)	0.055
*Clinical diagnosis*			
Stable angina	20 (36.4)	80 (36.4)	Matched
Unstable angina	6 (10.9)	24 (10.9)	Matched
NSTEMI	3 (5.5)	12 (5.5)	Matched
STEMI	26 (47.3)	104 (47.3)	Matched

Values are mean ± SD or *n* (%)

*ACS* acute coronary syndrome, *BMI* body-mass index, *GFR* glomerular filtration rate, *IDDM* insulin-dependent diabetes mellitus, *LVEF* left ventricular ejection fraction, *PAD* peripheral artery disease, *(N)STEMI* (non‑)ST-segment elevation myocardial infarction

**Table 2:** Procedural characteristics of patients with angiographically confirmed stent thrombosis and matched controls

	Angiographically confirmed ST (*n* = 55)	Matched controls (*n* = 220)	*p*-value
*Coronary anatomy*			
Left main artery	5 (9.1)	7 (3.2)	0.055
Left anterior descending artery	22 (40.0)	99 (45.0)	0.50
Circumflex artery	11 (20.0)	43 (19.5)	0.94
Right coronary artery	17 (30.9)	71 (32.3)	0.85
*Lesion characteristics*			
AHA/ACC lesion complexity B2 or C	49 (89.1)	114 (51.8)	<0.001
Chronic total occlusion	3 (5.5)	13 (5.9)	0.90
Bifurcation lesion	26 (44.8)	80 (34.2)	0.13
Ostial lesion	13 (23.6)	26 (11.8)	0.025
Severe calcification	20 (36.4)	72 (32.7)	0.61
Severe tortuosity	4 (7.0)	13 (5.9)	0.76
Visible thrombus	17 (30.9)	71 (32.3)	0.85
Eccentric coronary vessel	4 (6.9)	11 (4.7)	0.49
*Procedural characteristics*			
BMS implantation	26 (47.2)	88 (40.0)	0.29
DES implantation	29 (52.7)	132 (60.0)	0.25
First-generation DES	1 (1.8)	5 (2.3)	0.81
Second-generation DES	28 (48.3)	127 (56.2)	0.28
Mixed stents	2 (3.4)	5 (2.2)	0.58
No. of stents per patient	1.98 ± 0.82	1.56 ± 1.30	0.004
Stent length per patient, mm	38.2 ± 23.9	27.3 ± 16.0	<0.001
Stent diameter, mm	3.11 ± 10.4	2.96 ± 0.42	0.47
Pre-dilatation balloon pressure, atm	13.9 ± 4.3	11.8 ± 3.5	0.003
Stent implantation pressure, atm	14.5 ± 2.6	14.5 ± 3.3	0.98
Post-dilatation balloon pressure, atm	18.8 ± 3.6	18.4 ± 5.2	0.64
TIMI flow grade 3 before PCI	28 (50.9)	117(53.2)	0.77
TIMI flow grade 3 after PCI	51 (92.7)	218 (99.1)	0.004
Post procedural GpIIb/IIIa therapy	14 (25.5)	61 (27.7)	0.73

Values are mean ± SD or *n* (%)

*AHA/ACC* American Heart Association/American College of Cardiology,* BMS* bare-metal stent, *DES* drug-eluting stent, *PCI* percutaneous coronary intervention, *ST* stent thrombosis, *TIMI* thrombolysis in myocardial infarction

### Reassessment of coronary angiography

**Fig. 3:**
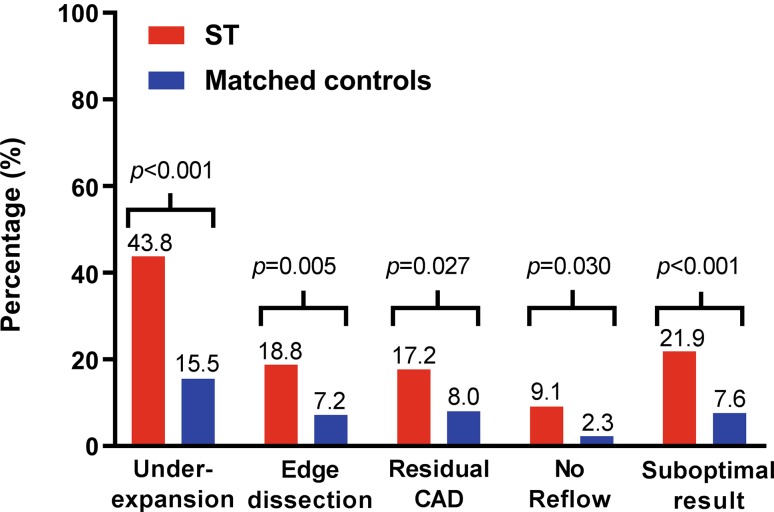
Frequencies of angiographic predictors and grading of the angiographic result for patients with stent thrombosis and matched controls

The inter-observer reliability for the overall angiographic reassessment was substantial (κ = 0.63, Supplementary Appendix). Reassessment of index angiograms showed a significant difference in angiographic stent underexpansion (43.8% vs 15.5%, *p* < 0.001), angiographic stent edge dissection (18.8% vs 7.2%, *p* = 0.005), residual coronary artery disease (CAD) (17.2% vs 8.0%, *p* = 0.027), and no-reflow phenomenon (9.1% vs 2.3%, *p* < 0.030) in ST as compared to the matched controls. Patients who suffered ST had a suboptimal angiographic result more often than the matched controls (21.9% vs 7.6%, *p* < 0.001) (Fig. 3).

### Impact of antiplatelet agents on ST

We were able to verify the use of antiplatelet agents and oral anticoagulation therapy in 216 of 275 patients (>75%). DAPT non-use was common in patients following PCI, as we identified 13 cases (23.6%) that were not taking DAPT prior to ST as compared to 5 (2.3%) of the matched controls (*p* < 0.001). In more detail, 2 of 55 (3.6%) cases did not receive a P2Y12 inhibitor unintentionally, 6 of 55 were non-compliant (10.9%), and in 5 of 55 (9.1%) patients DAPT was discontinued.

### Comprehensive identification of predictors of ST

Independent clinical, angiographic, and procedural predictors of angiographically confirmed ST were identified using a multivariable logistic regression model (Tab. 3). The strongest independent predictor of angiographically confirmed ST was DAPT non-use (OR 10.9, 95% CI 2.47–48.5, *p* < 0.001), followed by: stent underexpansion (OR 5.70, 95% CI 2.39–13.6, *p* < 0.001), lesion complexity B2/C (OR 4.32, 95% CI 1.43–13.1, *p* = 0.010), uncovered stent edge dissection (OR 4.16, 95% CI 1.47–11.8, *p* = 0.007), diabetes mellitus (OR 3.23, 95% CI 1.25–8.36, *p* = 0.016), and residual CAD 5 mm proximal or distal to the stent (OR 3.02, 95% CI 1.02–8.92, *p* = 0.045).

**Table 3:** Multivariable predictors of angiographically confirmed ST as compared to matched controls

	Univariable analysis			Multivariable analysis^a^		
	OR	95% CI	*p*-value	OR	95% CI	*p*-value
DAPT non-use	15.8	4.48 to 55.8	*p* < 0.001	10.9	2.47 to 48.5	*p* < 0.001
No-reflow phenomenon	5.36	1.25 to 23.0	*p* = 0.024	6.72	0.80 to 56.5	*p* = 0.079
Stent underexpansion	7.35	3.60 to 15.0	*p* < 0.001	5.70	2.39 to 13.6	*p* < 0.001
Lesion complexity B2 or C	8.24	3.18 to 21.4	*p* < 0.001	4.32	1.43 to 13.1	*p* = 0.010
Stent edge dissection	3.67	1.61 to 8.34	*p* = 0.002	4.16	1.47 to 11.8	*p* = 0.007
Diabetes mellitus	1.83	0.93 to 3.60	*p* = 0.079	3.32	1.25 to 8.36	*p* = 0.016
Residual CAD	2.73	1.18 to 6.31	*p* = 0.019	3.02	1.07 to 8.92	*p* = 0.045

^a^Variables in the conditional logistic regression model were: age, sex, body-mass index, diabetes mellitus, smoking status, left ventricular ejection fraction <35%, prior peripheral artery disease, prior acute coronary syndrome, prior malignancy, dual antiplatelet therapy (*DAPT*) non-use, lesion complexity B2/C, stent underexpansion, stent edge dissection, residual coronary artery disease (*CAD*), no-reflow phenomenon, impaired TIMI flow, and total stent length per patient

### Impact of ST on mortality

**Fig. 4:**
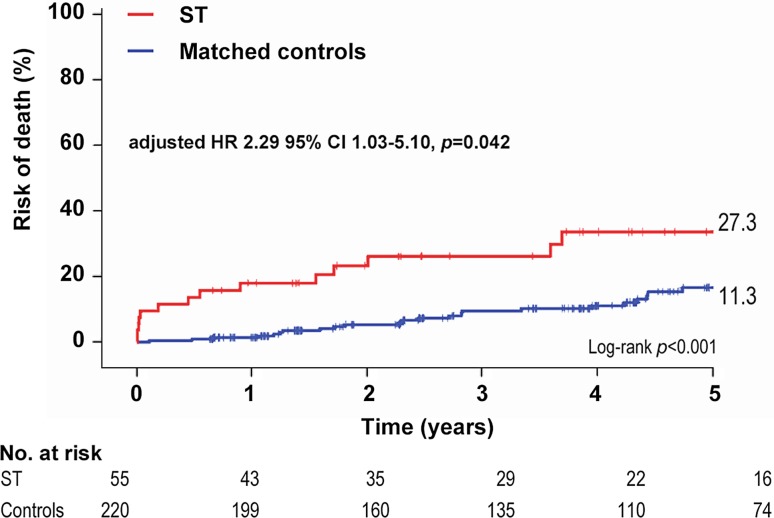
Kaplan-Meier estimates for the risk of death of angiographically confirmed stent thrombosis as compared to matched controls

Kaplan-Meier estimates of the cumulative risk of death were obtained for cases and matched controls with right censoring at 5 years’ follow-up (Fig. 4). Vital status was available for 273 patients (99.3%) with a median follow-up of 4.0 ± 2.1 years. Angiographically confirmed ST was associated with an increased risk of death as compared to the matched controls (27.3% vs 11.3%, *p*_log-rank_ < 0.001). No differences were found in mortality with respect to cases that were treated for acute PCI as compared to planned PCI (30.8% vs 24.1%, *p* = 0.31). After adjustments, ST patients had a two-fold increased risk of death as compared to matched controls (adjusted hazard ratio 2.29, 95% CI 1.03–5.10, *p* = 0.042).

## Discussion

The main findings of our study are that (1) patients who fail to use DAPT, patients with complex lesions, and patients with diabetes mellitus are at increased risk of ST; (2) a carefully executed reassessment of the coronary angiogram enabled us also to identify angiographic parameters that are strongly predictive for the risk of ST; and (3) ST continued to be a serious complication following PCI with a high rate of mortality, predominantly within the first year. Consistent with our findings, a large registry [8] found an incidence of definite ST of roughly 0.75–1.5% in the first year after implantation.

An important finding of our analysis was that 13 cases (23.6%) could be attributed to inadequate use of antiplatelet agents, supporting the fact that pharmacotherapy following PCI remains pivotal. This finding is consistent with those of most studies [2, 9–17], which showed that inadequate DAPT was the strongest predictor of ST, although the results of other studies [18, 19] failed to demonstrate this association. Especially in complex patients with angiographic stent underexpansion, dissections, or residual CAD, a more aggressive pharmacotherapeutic strategy (i. e. longer regimens of DAPT, high-potency lipid-lowering or antihypertensive agents) or counselling on DAPT compliance may be considered in selected patients.

Angiographic stent underexpansion, lesion complexity, uncovered edge dissection, and residual CAD within 5 mm of the stent edge were associated with ST. Various studies have addressed the importance of adequate stent deployment [20] and sizing [21], particularly in patients with complex lesions. Studies employing intravascular ultrasound [22, 23] and optical coherence tomography [24–26] indicate that inadequate stent expansion ranges from 20 to 40% and stent edge dissection ranges from 10 to 50%. Based on our findings over 40% of the lesions in patients who suffered ST were classified as angiographically underexpanded, whereas the final angiographic result was optimal in nearly 80% of these patients, emphasising the complexity of the lesions in patients prone to ST. Our findings are consistent with those of the ADAPT-DES study [27], which reports that IVUS-guided PCI made the operator choose higher inflation pressures (23%), additional post-dilatation for underexpansion (20%), additional stent placement (8%), or longer stents (22%). These reconsiderations reduced the 1‑year rate of ST by 0.5%. Based on our findings, longer DES may be considered to prevent residual CAD. From a practical point of view, it would be interesting to investigate if a careful angiographic re-evaluation may establish a reduction in adverse clinical outcomes in selected patients.

Consistent with previous reports [28–30], roughly 90% of cases of definite ST occurred in patients with complex coronary lesions. This could have several possible explanations: (1) complex lesions enhance the risk of delayed or incomplete stent strut endothelialisation or stent malapposition, which could trigger platelet activation; (2) in patients undergoing complex PCI, more stents and longer stents are implanted, both increasing the chance of thrombogeneity and potentially predisposing to the occurrence of ST; (3) patients who underwent complex PCI could have more advanced CAD and more comorbidities that lead to faster disease progression and sudden onset of alterations that make them prone to ST.

Previous reports [31, 32] show considerable variation in mortality ranging from 17 to 45%, dependent on clinical indication and timing of ST. Given the high risk of mortality that is associated with ST, this study emphasises the importance of ongoing efforts to identify modifiable predictors of this potentially devastating complication.

### Study limitations

This study has some limitations that should be acknowledged. First, by definition we did not incorporate cases of very late ST that may have underestimated the cumulative incidence by approximately 15%. Moreover, we did not consider ST from: (1) sudden cardiac death or (2) silent stent occlusion. Second, although no independent angiographic core laboratory was consulted, angiograms were reassessed and quantified in a structured, blinded, and reliable manner. We believe that the lack of an independent core laboratory did not affect the main results of this study. Third, even though we consider verified DAPT usage from linking prescription data to pharmacy records as a strength of our analysis, we obtained complete data in roughly three-quarters of our control patients that may have introduced a certain degree of bias that we tried to minimise by using data imputation. Finally, our study reflects an analysis of BMS and DES implantations, given the time period during which it was conducted. However, since BMS are obsolete in current practice, it would have been interesting to study DES exclusively.

## Conclusion

Definite stent thrombosis continues to be a serious complication following stent implantation and is associated with high rates of mortality. DAPT non-use was associated with the highest risk of ST, followed by angiographic stent underexpansion, lesion complexity B2/C, uncovered stent edge dissection, residual coronary artery, and diabetes mellitus. Angiographic parameters are important to define an individual patient risk of ST.

## Caption Electronic Supplementary Material

Supplementary Materials on Reassessment of the coronary angiograms

## References

[1] Daemen J, Wenaweser P, Tsuchida K (2007). Early and late coronary stent thrombosis of sirolimus-eluting and paclitaxel-eluting stents in routine clinical practice: data from a large two-institutional cohort study. Lancet.

[2] Kuchulakanti PK, Chu WW, Torguson R (2006). Correlates and long-term outcomes of angiographically proven stent thrombosis with sirolimus- and paclitaxel-eluting stents. Circulation.

[3] Garcia-Garcia HM, McFadden EP, Farb A (2018). Standardized end point definitions for coronary intervention trials: the Academic Research Consortium-2 consensus document. Circulation.

[4] Bland JM, Altman DG (1986). Statistical methods for assessing agreement between two methods of clinical measurement. Lancet.

[5] Ellis SG, Vandormael MG, Cowley MJ (1990). Coronary morphologic and clinical determinants of procedural outcome with angioplasty for multivessel coronary disease. Implications for patient selection. Multivessel Angioplasty Prognosis Study Group. Circulation.

[6] Sterne JA, White IR, Carlin JB (2009). Multiple imputation for missing data in epidemiological and clinical research: potential and pitfalls. BMJ.

[7] Pocock SJ, Clayton TC, Altman DG (2002). Survival plots of time-to-event outcomes in clinical trials: good practice and pitfalls. Lancet.

[8] Tada T, Byrne RA, Simunovic I (2013). Risk of stent thrombosis among bare-metal stents, first-generation drug-eluting stents, and second-generation drug-eluting stents: results from a registry of 18,334 patients. JACC Cardiovasc. Interv..

[9] Spertus JA, Kettelkamp R, Vance C (2006). Prevalence, predictors, and outcomes of premature discontinuation of thienopyridine therapy after drug-eluting stent placement: results from the PREMIER registry. Circulation.

[10] Airoldi F, Colombo A, Morici N (2007). Incidence and predictors of drug-eluting stent thrombosis during and after discontinuation of thienopyridine treatment. Circulation.

[11] van Werkum JW, Heestermans AA, Zomer AC (2009). Predictors of coronary stent thrombosis: the Dutch Stent Thrombosis Registry. J Am Coll Cardiol.

[12] Ferreira-Gonzalez I, Marsal JR, Ribera A (2010). Background, incidence, and predictors of antiplatelet therapy discontinuation during the first year after drug-eluting stent implantation. Circulation.

[13] Mehran R, Baber U, Steg PG (2013). Cessation of dual antiplatelet treatment and cardiac events after percutaneous coronary intervention (PARIS): 2 year results from a prospective observational study. Lancet.

[14] de la Torre-Hernandez JM, Alfonso F, Hernandez F (2008). Drug-eluting stent thrombosis: results from the multicenter Spanish registry ESTROFA (Estudio ESpanol sobre TROmbosis de stents FArmacoactivos). J Am Coll Cardiol.

[15] Chechi T, Vecchio S, Vittori G (2008). ST-segment elevation myocardial infarction due to early and late stent thrombosis a new group of high-risk patients. J Am Coll Cardiol.

[16] Wenaweser P, Rey C, Eberli FR (2005). Stent thrombosis following bare-metal stent implantation: success of emergency percutaneous coronary intervention and predictors of adverse outcome. Eur Heart J.

[17] Aoki J, Lansky AJ, Mehran R (2009). Early stent thrombosis in patients with acute coronary syndromes treated with drug-eluting and bare metal stents: the Acute Catheterization and Urgent Intervention Triage Strategy Trial. Circulation.

[18] Piccolo R, Feres F, Abizaid A (2017). Risk of early adverse events after clopidogrel discontinuation in patients undergoing short-term dual antiplatelet therapy: an individual participant data analysis. JACC Cardiovasc. Interv..

[19] Kimura T, Morimoto T, Nakagawa Y (2009). Antiplatelet therapy and stent thrombosis after sirolimus-eluting stent implantation. Circulation.

[20] Ali ZA, Maehara A, Genereux P (2016). Optical coherence tomography compared with intravascular ultrasound and with angiography to guide coronary stent implantation (ILUMIEN III: OPTIMIZE PCI): a randomised controlled trial. Lancet.

[21] Sera F, Awata M, Uematsu M (2009). Optimal stent-sizing with intravascular ultrasound contributes to complete neointimal coverage after sirolimus-eluting stent implantation assessed by angioscopy. JACC Cardiovasc. Interv..

[22] Claessen BE, Mehran R, Mintz GS (2011). Impact of intravascular ultrasound imaging on early and late clinical outcomes following percutaneous coronary intervention with drug-eluting stents. JACC Cardiovasc. Interv..

[23] Hong SJ, Kim BK, Shin DH (2015). Effect of intravascular ultrasound-guided vs angiography-guided everolimus-eluting stent implantation: the IVUS-XPL randomized clinical trial. JAMA.

[24] Wijns W, Shite J, Jones MR (2015). Optical coherence tomography imaging during percutaneous coronary intervention impacts physician decision-making: ILUMIEN I study. Eur Heart J.

[25] Souteyrand G, Amabile N, Mangin L (2016). Mechanisms of stent thrombosis analysed by optical coherence tomography: insights from the national PESTO French registry. Eur Heart J.

[26] Prati F, Kodama T, Romagnoli E (2015). Suboptimal stent deployment is associated with subacute stent thrombosis: optical coherence tomography insights from a multicenter matched study. From the CLI Foundation investigators: the CLI-THRO study. Am Heart J.

[27] Witzenbichler B, Maehara A, Weisz G (2014). Relationship between intravascular ultrasound guidance and clinical outcomes after drug-eluting stents: the assessment of dual antiplatelet therapy with drug-eluting stents (ADAPT-DES) study. Circulation.

[28] Genereux P, Redfors B, Witzenbichler B (2017). Angiographic predictors of 2‑year stent thrombosis in patients receiving drug-eluting stents: insights from the ADAPT-DES study. Catheter Cardiovasc Interv.

[29] Cayla G, Hulot JS, O’Connor SA (2011). Clinical, angiographic, and genetic factors associated with early coronary stent thrombosis. JAMA.

[30] Giustino G, Chieffo A, Palmerini T (2016). Efficacy and safety of dual antiplatelet therapy after complex PCI. J Am Coll Cardiol.

[31] Iakovou I, Schmidt T, Bonizzoni E (2005). Incidence, predictors, and outcome of thrombosis after successful implantation of drug-eluting stents. JAMA.

[32] Ergelen M, Gorgulu S, Uyarel H (2010). The outcome of primary percutaneous coronary intervention for stent thrombosis causing ST-elevation myocardial infarction. Am Heart J.

