# Supplementary material for: Incidence, angiographic and clinical predictors, and impact of stent thrombosis: a 6-year survey of 6,545 consecutive patients
Source: Neth Heart J. 2019 Mar 20;27(6):321–9. doi: 10.1007/s12471-019-1253-2 (PMC6533324; doi:10.1007/s12471-019-1253-2)
Supplement: Supplementary file 1 — Supplementary Materials on Reassessment of the coronary angiograms [file 12471_2019_1253_MOESM1_ESM.docx]

**Supplementary Methods**

**1.1 Reassessment of Coronary Angiography**

Coronary angiography (CAG) reassessment was conducted using Xcelera software (Philips Healthcare, Best, The Netherlands). Baseline and post-PCI angiograms of the index procedures were individually reassessed in random order by two well-experienced interventional cardiologists whom were blinded to the objective of the study, and to patient outcome. In case of disagreement consensus was reached by a third interventional cardiologist. A complete list of the angiographic definitions used in our study is provided in Supplementary Appendix A. In brief, angiographic stent under expansion was defined as (1) the index-stent not being fully deployed, or (2) inappropriate alignment of the stent with the vessel wall, or (3) a mismatch in post-deployment stent dimensions in relation to the proximal/distal coronary segment. Angiographic uncovered stent edge dissection was defined as considerable haziness on the proximal/distal stent edge within 5 mm in the absence of calcifications. Residual coronary artery disease (CAD) was defined as significant disease within 5 mm of either stent edge with visually estimated percentage stenosis of ≥50%. Finally, the angiographic result was scored as: optimal (i.e. result could not be optimized), or suboptimal (i.e. result requires additional treatment). Subsequently, the level of inter-observer reliability[^6^](#_ENREF_6) (Cohen's kappa coefficient, κ) was calculated. Cohen's kappa coefficient ranges from −1.0 to +1.0, and was considered: poor (κ<0), slight (κ=0 to 0.20), fair (κ=0.21 to 0.40), moderate (κ=0.41 to 0.60), or substantial (κ=0.61 to 0.80).

Lesion complexity was defined according to the modified ACC/AHA lesion-specific classification[^7^](#_ENREF_7). Visible thrombus was defined as a contrast-filling defect on angiography in multiple projections. Severe coronary tortuosity was classified as: ≥1 bend(s) of≥90°, or if ≥3 bends of 45° to 90° proximal of the diseased segment. Severe lesion calcification was classified as: radiopacities noted without cardiac motion before contrast injection bilateral of the arterial lumen. Myocardial perfusion was scored before and after PCI, as defined by TIMI definitions[^8^](#_ENREF_8).

**1.2 List of Coronary Angiographic Definitions**

1. Angiographic Stent Under Expansion was defined as:
   1. index-stent not being fully deployed, or
   2. inappropriate alignment of the stent with the vessel wall, or
   3. a mismatch in post-deployment stent dimensions in relation to the proximal/distal coronary segment.
2. Angiographic Uncovered Stent Edge Dissection was defined as:
   1. considerable haziness on the proximal/distal stent edge within 5 mm, in the absence of calcifications.
3. Angiographic Residual Coronary Artery Disease was defined as:
   1. disease within 5 mm of either stent edge with visually estimated percentage stenosis of ≥50%.
4. A no-reflow Phenomenon was defined as:
   1. angiographic evidence of reopening of occluded coronary artery and successful stent placement with no evidence of flow-limiting residual stenosis, dissection, spasm, or apparent thrombus, and
   2. angiographic TIMI flow grade≤2, or a TIMI flow grade 3 with a TIMI myocardial perfusion grade 0 or 1, at least 5-10 min after PCI.
5. The Angiographic Result was scored as:
   1. ‘optimal’: treatment could not be optimized, or
   2. ‘suboptimal’: result requires additional treatment.
6. Angiographic Success was defined as:
   1. Coronary lesion treated with no residual stenosis of ≥20%, and TIMI flow grade 3.
7. Coronary Lesion Complexity(1) was defined according to the modified ACC/AHA lesion-specific into class A, B1, B2, or C:
8. Class A: Discrete (<10 mm length) Little or no calcification, concentric less than totally occlusive, readily accessible, nonangulated segment, <45°, smooth contour, not ostial in location, no major branch involvement, and absence of thrombus.
9. Class B1: a tubular (10-20 mm length), with at least one of the following: eccentric, moderate tortuosity of proximal segment, moderately angulated, segment, >45° <90°, moderate to severe calcification, total occlusion <3 months old, ostial in location, bifurcation lesions requiring double guide wires, irregular contour, some thrombus present.
10. Class B2: a tubular (10-20 mm length), two or more of the following: eccentric, moderate tortuosity of proximal segment, moderately angulated segment >45° <90°, moderate to severe calcification, total occlusion <3 months old, ostial in location, bifurcation lesions requiring double guide wires, irregular contour, some thrombus present.
11. Class C: Diffuse (>2 cm length), excessive tortuosity of proximal segment, extremely angulated, segments >90°, total occlusion >3 months old, inability to protect major side, branches, degenerated vein grafts with friable lesions.
12. Angiographic Visible Thrombus was defined as:
    1. Spheric, ovoid or irregular intraluminal filling defect or lucency surrounded on three sides by contrast medium seen just distal or within the coronary stenosis in multiple projections or a visible embolization of intraluminal material downstream.
13. Severe Calcification(2) was defined as:
    1. Severe lesion calcification was classified as: radiopacities noted without cardiac motion before contrast injection bilateral of the arterial lumen.
14. Sever Tortuosity
    1. One or more bends of 90° or more, or three or more bends of 45° to 90° proximal of the diseased segment.
15. Bifurcation lesion was defined as:
    1. A bifurcation is a division of a main, parent, branch into two daughter branches of at least 1.5mm. Bifurcation lesions may involve the proximal main vessel, the distal main vessel and the side branch according to the Medina classification. The smaller of the two daughter branches should be designated as the ‘side branch’. In case of the main stem either the LCX or the LAD can be designated as the side branch depending on their respective calibers. Bifurcations are scored for the following segment junctions: 5/6/11, 6/7/9, 7/8/10, 11/13/12a, 13/14/14a, 3/4/16 and 13/14/15.
16. Trifurcation lesion was defined as:
    1. A trifurcation is a division of a main branch into three branches of at least 1.5mm. Trifurcations are only scored for the following segment junctions: 3/4/16/16a, 5/6/11/12, 11/12a/12b/13, 6/7/9/9a and 7/8/10/10a.
17. Diffuse coronary artery disease was defined as:
    1. Present when at least 75% of the length of any segment(s) proximal to the lesion, at the site of the lesion or distal to the lesion has a vessel diameter of
18. Chronic Total Occlusion was defined as:
    1. No intra-luminal antegrade flow (TIMI 0) beyond the point of occlusion. However, antegrade flow beyond the total occlusion might be maintained by bridging collaterals and/or ipsi-collaterals.
19. Ostial lesion was defined as:
    1. A lesion is classified as aorto-ostial when it is located within 3 mm of the origin of the coronary vessels from the aorta (applies only to segments 1 and 5, or to 6 and 11 in case of double ostium of the LCA). or within 3-5 mm of the origin of a major epicardial artery.
20. Myocardial perfusion was scored before and after PCI, as defined by TIMI definitions(3).
21. Thrombolysis in Myocardial Infarction Coronary Flow:(4)
    1. TIMI 0 flow (no perfusion) refers to the absence of any antegrade flow beyond a coronary occlusion.
    2. TIMI 1 flow (penetration without perfusion) is faint antegrade coronary flow beyond the occlusion, with incomplete filling of the distal coronary bed.
    3. TIMI 2 flow (partial reperfusion) is delayed or sluggish antegrade flow with complete filling of the distal territory.
    4. TIMI 3 is normal flow which fills the distal coronary bed completely

**Supplementary Results**

**2.1 Reassessment of Coronary Angiography**

Inter-observer reliability is shown in figure 1. Overall the angiographic reassessment was substantial (κ=0.63), especially for angiographic stent under expansion (κ=0.61) and angiographic uncovered stent edge dissection (κ=0.73). The inter-observer reliability for residual CAD proximal or distal to the stent was moderate (κ=0.52).

Figure 1. inter-observer reliability for the angiographic reassessment.

**
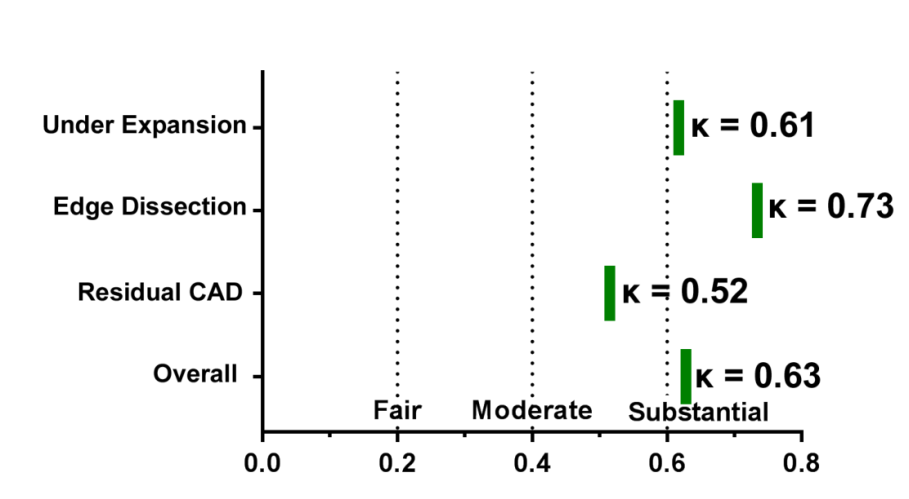
**

**References**

1. Ellis SG, Vandormael MG, Cowley MJ, DiSciascio G, Deligonul U, Topol EJ, et al. Coronary morphologic and clinical determinants of procedural outcome with angioplasty for multivessel coronary disease. Implications for patient selection. Multivessel Angioplasty Prognosis Study Group. Circulation. 1990;82(4):1193-202.

2. Madhavan MV, Tarigopula M, Mintz GS, Maehara A, Stone GW, Genereux P. Coronary artery calcification: pathogenesis and prognostic implications. J Am Coll Cardiol. 2014;63(17):1703-14.

3. Group TS. The Thrombolysis in Myocardial Infarction (TIMI) trial. Phase I findings. N Engl J Med. 1985;312(14):932-6.

4. Holmes DR, Jr., Holubkov R, Vlietstra RE, Kelsey SF, Reeder GS, Dorros G, et al. Comparison of complications during percutaneous transluminal coronary angioplasty from 1977 to 1981 and from 1985 to 1986: the National Heart, Lung, and Blood Institute Percutaneous Transluminal Coronary Angioplasty Registry. J Am Coll Cardiol. 1988;12(5):1149-55.
